# Supplementary material for: Shared EEG correlates between non-REM parasomnia experiences and dreams
Source: Nat Commun. 2024 May 9;15:3906. doi: 10.1038/s41467-024-48337-7 (PMC11082195; doi:10.1038/s41467-024-48337-7)
Supplement: Supplementary file 1 — Supplementary Information [file 41467_2024_48337_MOESM1_ESM.docx]

Figure S1**
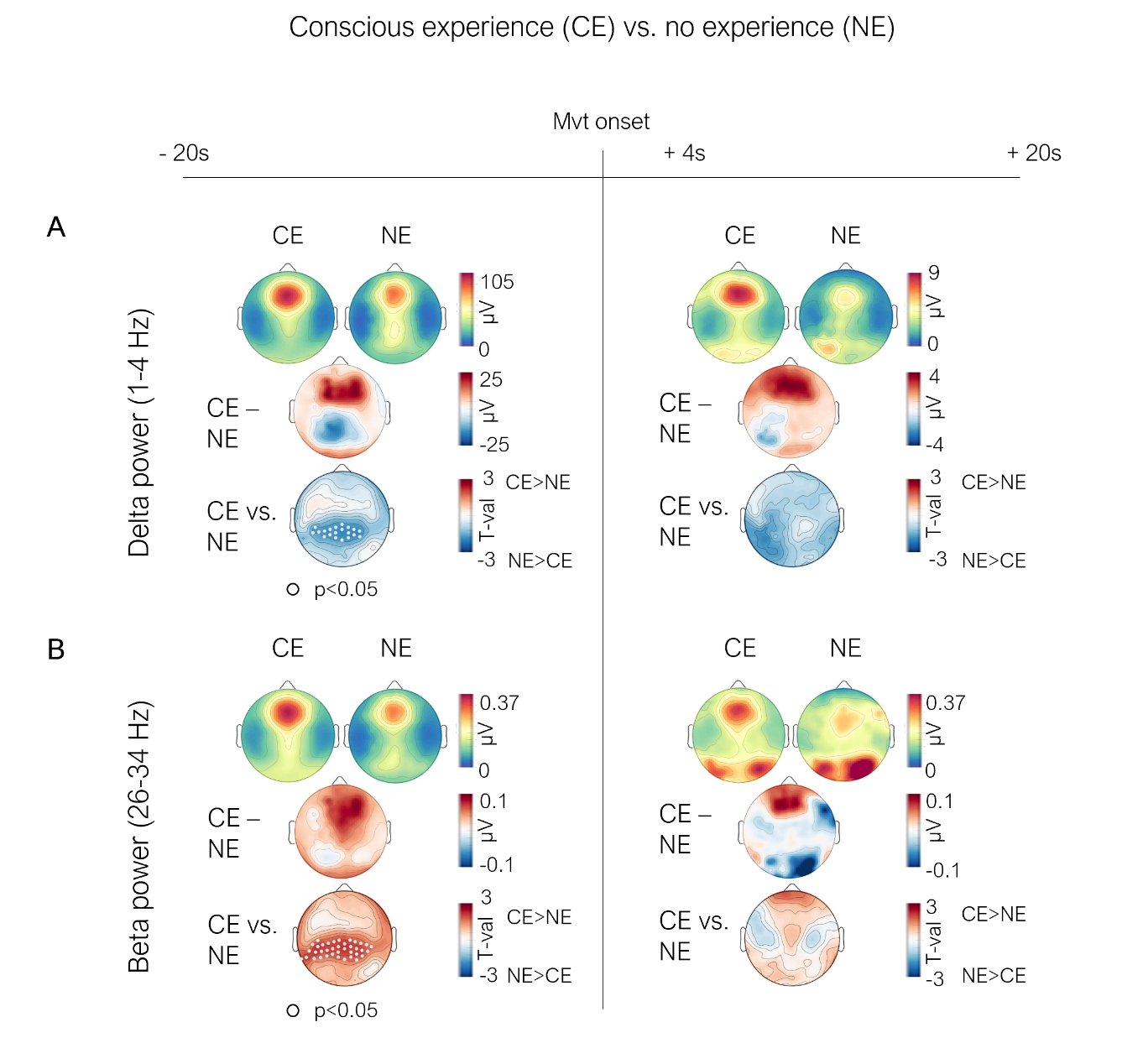
**

Fig. S1: Conscious experience (CE) vs. no experience (NE): spectral power scalp level. A. Left column: topographical distribution of

absolute delta power (1-4 Hz) averaged across subjects for CE and NE, of the absolute difference (CE minus NE), and of t-values

[Wald statistics, CE (n = 32 episodes from 14 participants) vs. NE (n = 11 episodes from 7 participants )] at the scalp level for the 20

seconds of sleep preceding movement onset. Right; same as left for the period from 4 to 20 seconds after movement onset CE (n = 31 episodes from 14 participants) vs. NE (n = 10 episodes from 7 participants). 175 innermost channels are displayed. B. Same as A for beta power (26 – 34 Hz). Mvt onset= movement onset.

Figure S2

**
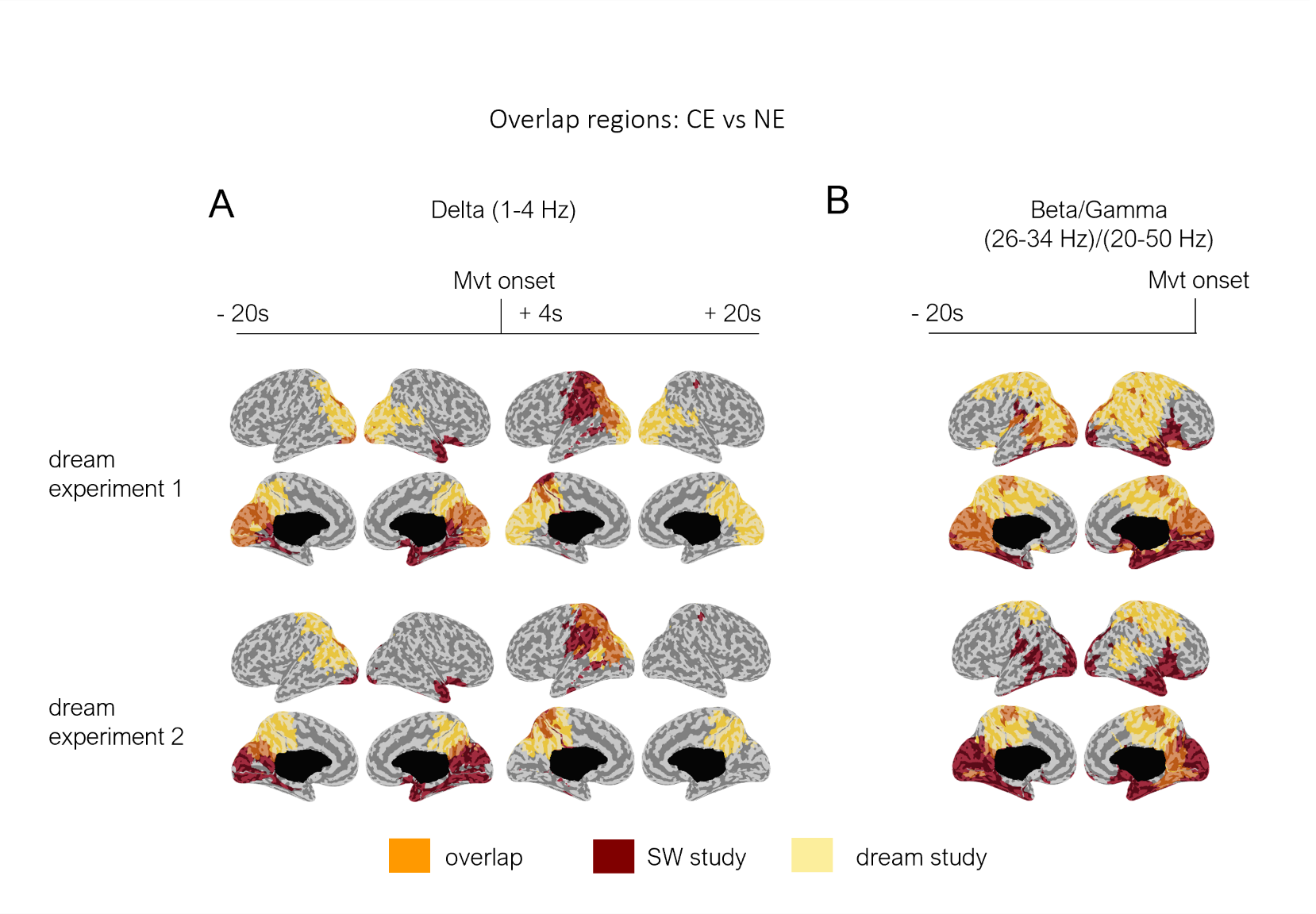
**

Fig. S2: Conjunction maps: differences and overlap between the contrasts ‘conscious experience/no experience’ in the current study (SW study) and the contrast ‘dream experience/no experience’ in two previous experiments on the neural correlates of dreaming in healthy participants (dream study)^1^ for low frequency power (A) and high-frequency power (B). Power in the high-frequencies was analyzed in the beta band in the current study (26-34 Hz), and in the gamma band (20-50Hz) in the previous study on dreaming. Statistics were not computed in the same manner (generalized linear mixed models in the current study, paired t-tests in the dream study).


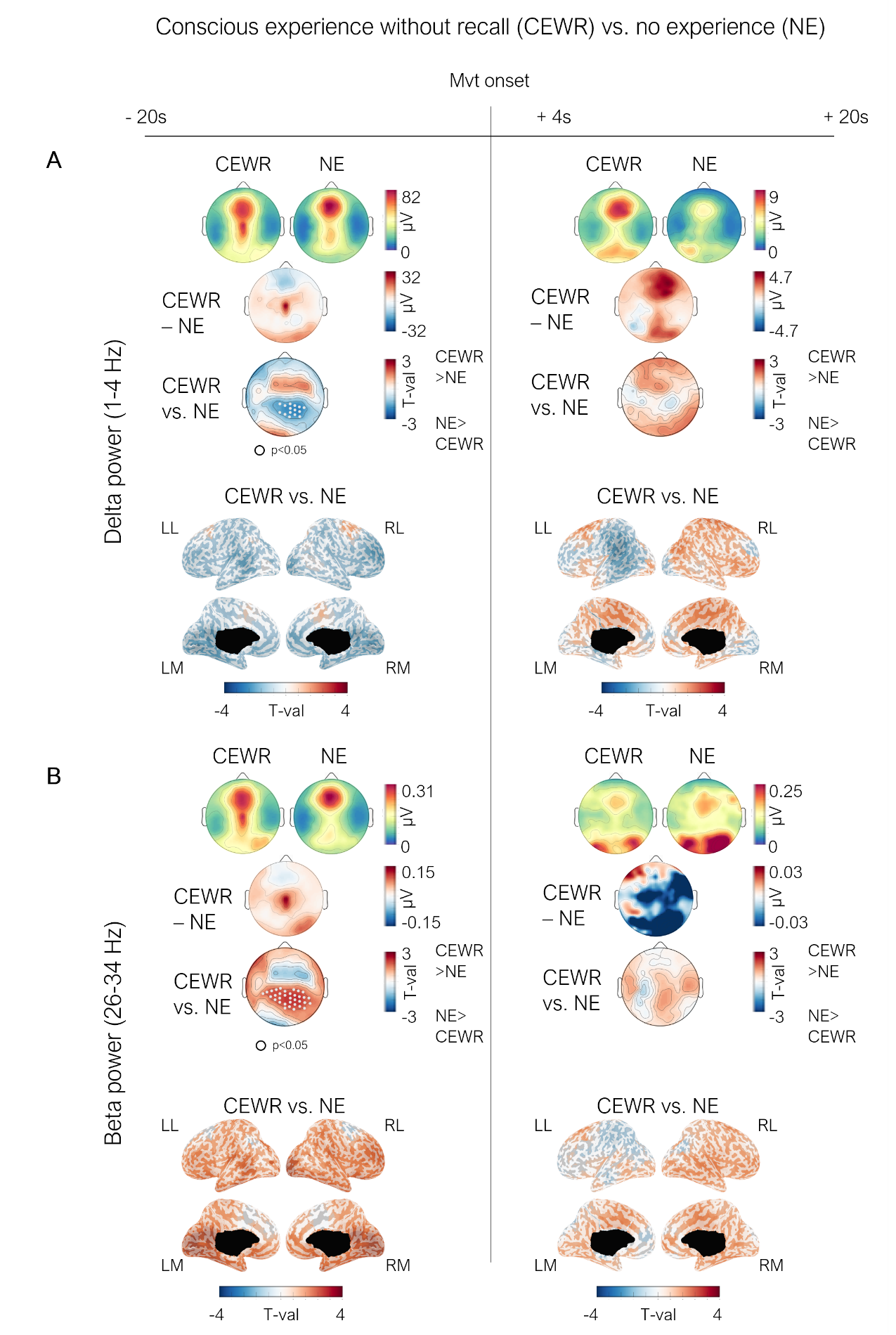
Figure S3

Fig. S3: Conscious experience without recall of content (CEWR) vs. no experience (NE): spectral power. A. Left column: topographical

distribution of absolute sleep delta power (1-4 Hz) averaged across subjects for CEWR and NE, of the absolute difference (CEWR

minus NE), and of t-values [Wald statistics, CEWR (n = 17 episodes from 8 participants) vs. NE (n = 11 episodes from 7 participants)]

at the scalp and source level for 20 seconds of sleep preceding movement onset. Right; same as left for the period from 4 to 20

seconds after movement onset [CEWR (n = 17 episodes from 8 participants) vs. NE (n = 10 episodes from 7 participants)].

175 innermost channels are displayed at the scalp level. LL, left lateral; RL, right lateral; LM, left medial; RM, right medial view. B. Same as A for beta power (26–34 Hz). Mvt onset= movement onset.


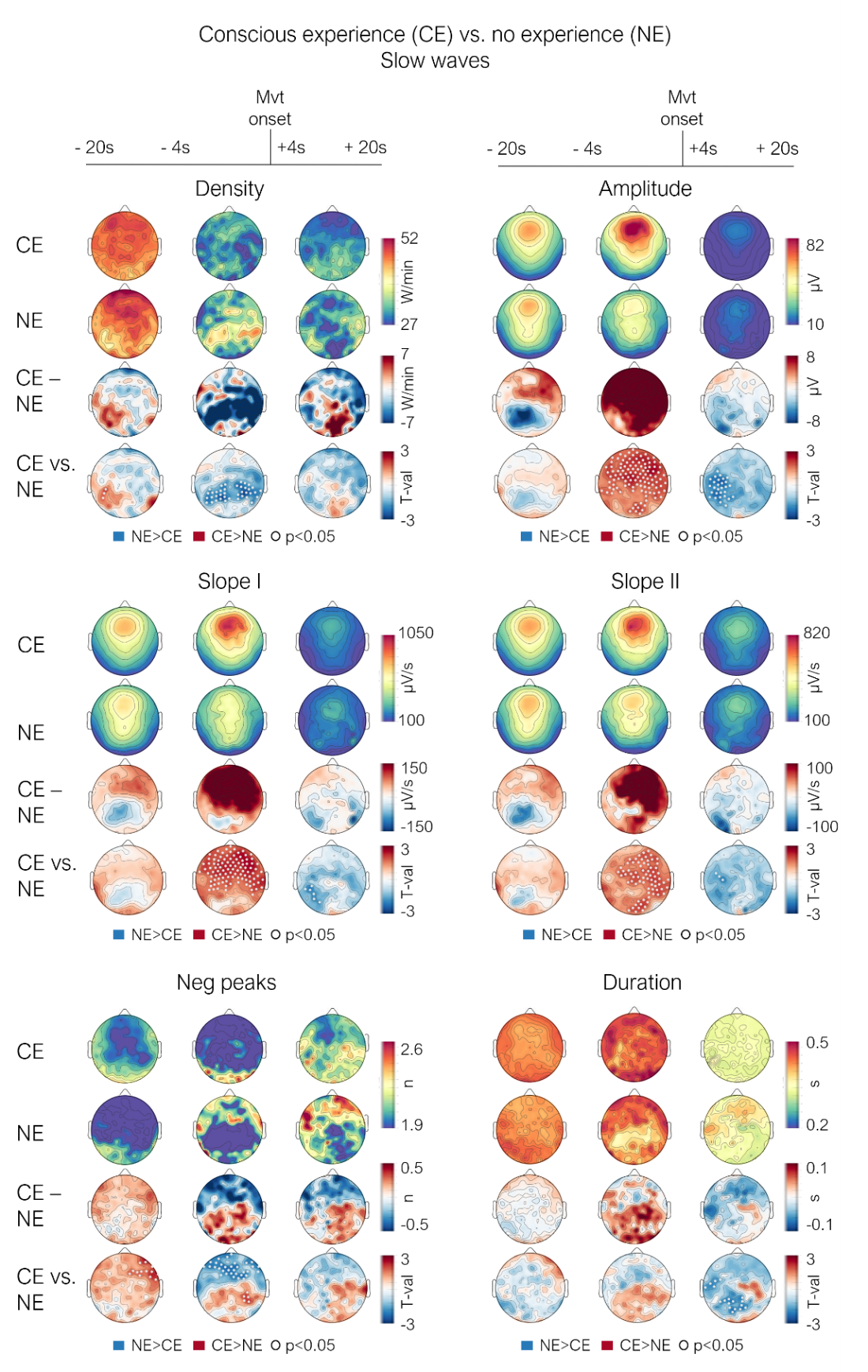
Figure S4

Fig.S4: Conscious experience (CE) vs. no experience (NE): slow wave parameters. Topographical distribution of slow wave

parameters averaged across subjects for CE and NE, of their absolute difference (CE minus NE), and of t-values [Wald statistics, CE

(n = 32 episodes from 14 participants) vs. NE (n = 11 episodes from 7 participants)] for three timeframes [from -20 to -4s before

movement onset CE (n = 32 episodes from 14 participants) vs. NE (n = 11 episodes from 7 participants), from -4s to movement onset

CE (n = 32 episodes from14 participants) vs. NE (n = 11 episodes from 7 participants) and from +4s to +20s CE (n = 31 episodes from

14 participants) vs. NE (n = 10 episodes from 7 participants)]. 175 innermost channels are displayed. Density=number of slow waves per minute, Amplitude= absolute amplitude of maximum negative slow wave peak. Slope I=positive to negative deflection of the slow wave, Slope II=slope of negative to positive deflection of slow wave, Neg Peaks= number of negative intrawave peaks, Duration = time between two zero-line crossings. Mvt onset= movement onset.

Figure S5

**
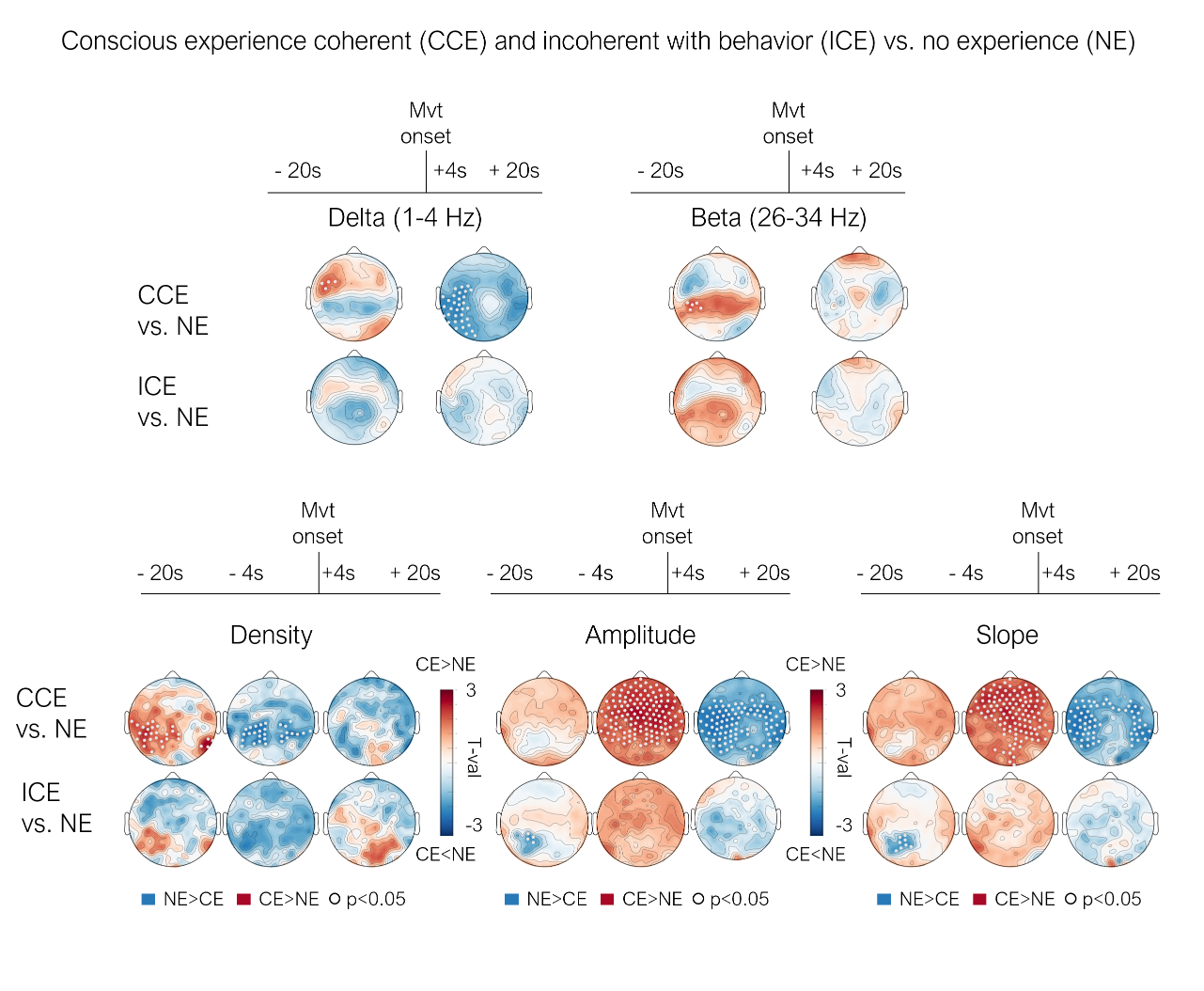
**

Fig. S5: Coherent (CCE) and incoherent (ICE) conscious experiences vs. no experience (NE). A. Topographical distribution of statistical differences in delta and beta power (t-values, Wald statistics) for CCE vs. NE (top row in each panel; n=15 episodes from 9 participants vs. n = 11 episodes from 7 participants) and ICE vs. NE (bottom row in each panel; n=13 episodes from 9 participants vs n = 11 episodes from 7 participants), shown for the EEG corresponding to sleep (left) and parasomnia episode (right). Topographical distribution of statistical differences in slow wave parameters (t-values, Wald statistics) for CCE vs. NE (top row in each panel; n=15 episodes from 9 participants vs. 11 episodes from 7 participants) and ICE vs. NE (bottom row in each panel; n=13 episodes from 9 participants vs 11 episodes from 7 participants) shown for three timeframes (sleep from -20 to -4s before movement onset, from -4s to movement onset and from +4s to +20s after movement onset).175 innermost channels are displayed. Density=number of slow waves per minute, Amplitude= absolute amplitude of maximum negative slow wave peak, Slope =slope of negative to positive deflection of slow wave. Mvt onset= movement onset.


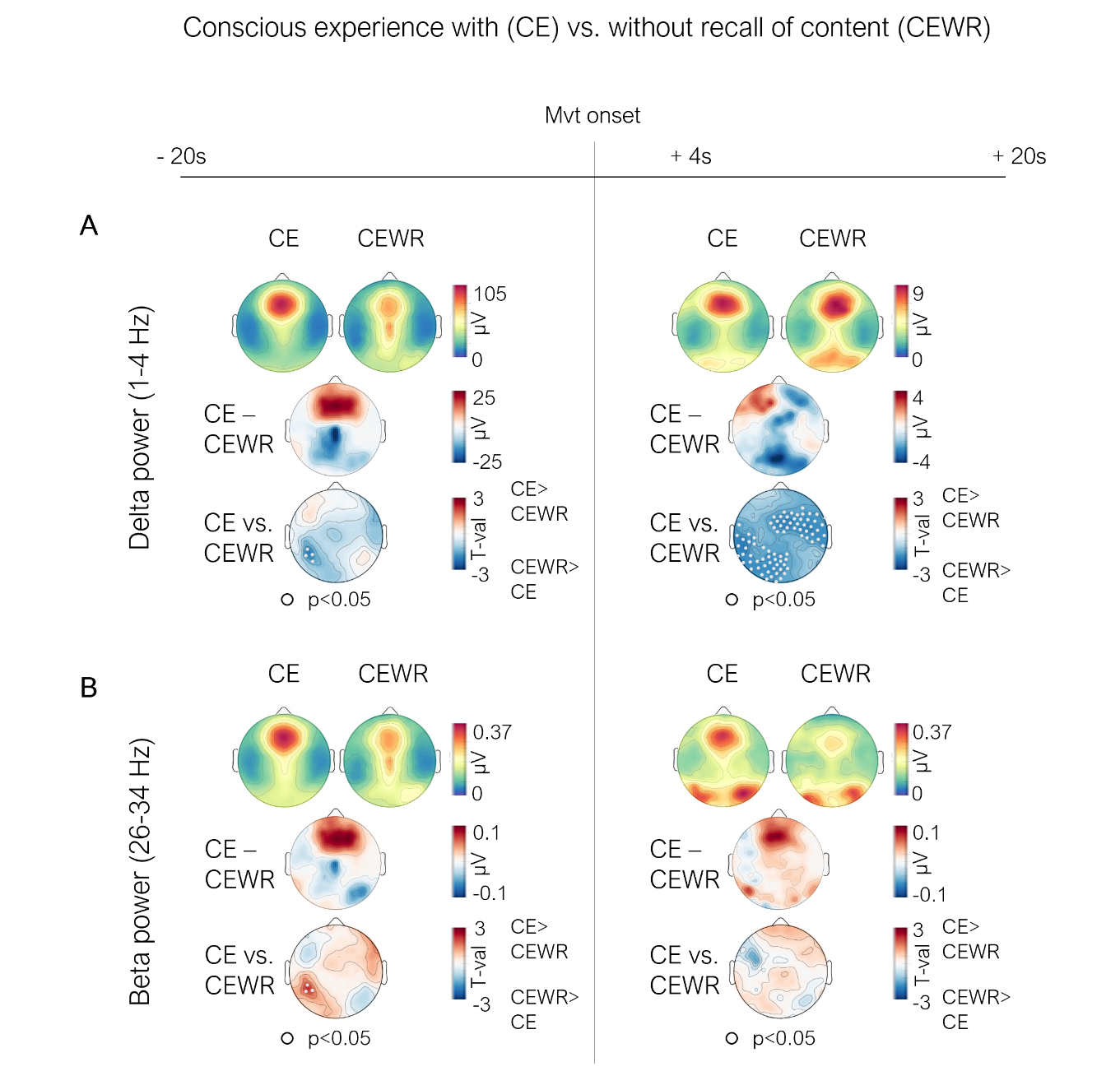
Figure S6

Fig. S6. Conscious experience with (CE) vs. without recall of content (CEWR). A. Left column: topographical distribution of absolute

sleep delta power (1-4 Hz) averaged across subjects for CE and CEWR of the absolute difference (CE minus CEWR), and of t-values

[Wald statistics, CE (n = 32 episodes from 14 participants) vs. CEWR (n = 17 episodes from 8 participants)] at the scalp and source

level for 20 seconds of sleep preceding movement onset. Right; same as left for the period from 4 to 20 seconds after movement

onset CE (n = 31 episodes from 14 participants) vs. CEWR (n = 17 episodes from 8 participants). 175 innermost channels are

displayed at the scalp level. LL, left lateral; RL, right lateral; LM, left medial; RM, right medial view. B. Same as A for beta power (26–34 Hz).


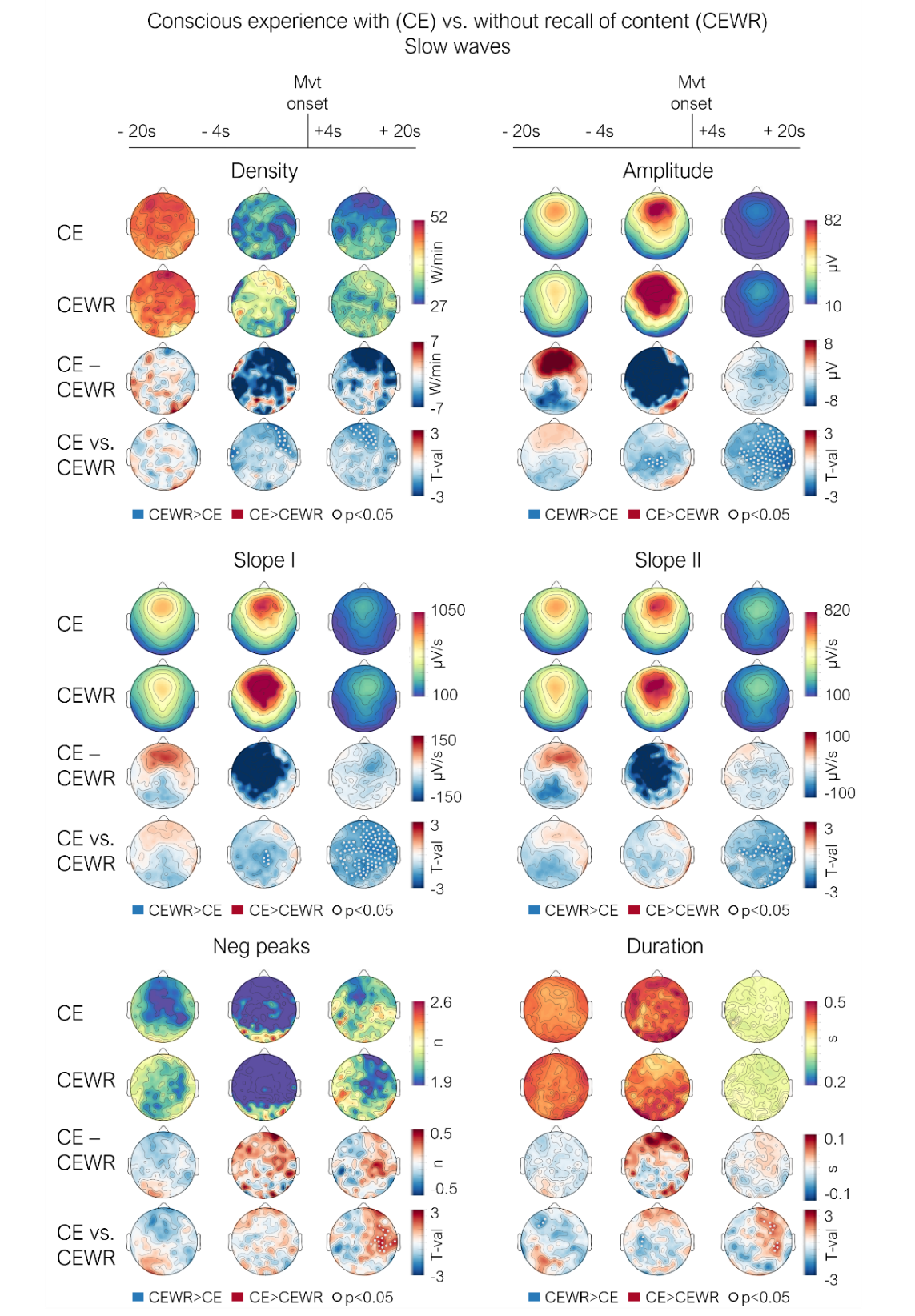
Figure S7

Fig. S7: Conscious experience with (CE) vs. without recall (CEWR): slow wave parameters. Topographical distribution of slow wave

parameters averaged across subjects for CE and CEWR, of their absolute difference (CE minus CEWR), and of t-values [Wald

statistics, CE (n = 32 episodes from 14 participants) vs. CEWR (n = 17 episodes from 8 participants)] for three timeframes [from -20 to

-4s before movement onset CE (n = 32 episodes from14 participants) vs. CEWR (n = 17 episodes from 8 participants), from -4s to

movement onset CE (n = 32 episodes from14 participants) vs. CEWR (n = 17 episodes from 8 participants) and from +4s to +20s CE

(n = 31 episodes from 14 participants) vs. CEWR (n = 17 episodes from 8 participants)]. 175 innermost channels are displayed. Density=number of slow waves per minute, Amplitude= absolute amplitude of maximum negative slow wave peak. Slope I=positive to negative deflection of the slow wave, Slope II=slope of negative to positive deflection of slow wave, Neg Peaks= number of negative intrawave peaks, Duration = time between two zero-line crossings. Mvt onset= movement onset.

Figure S8


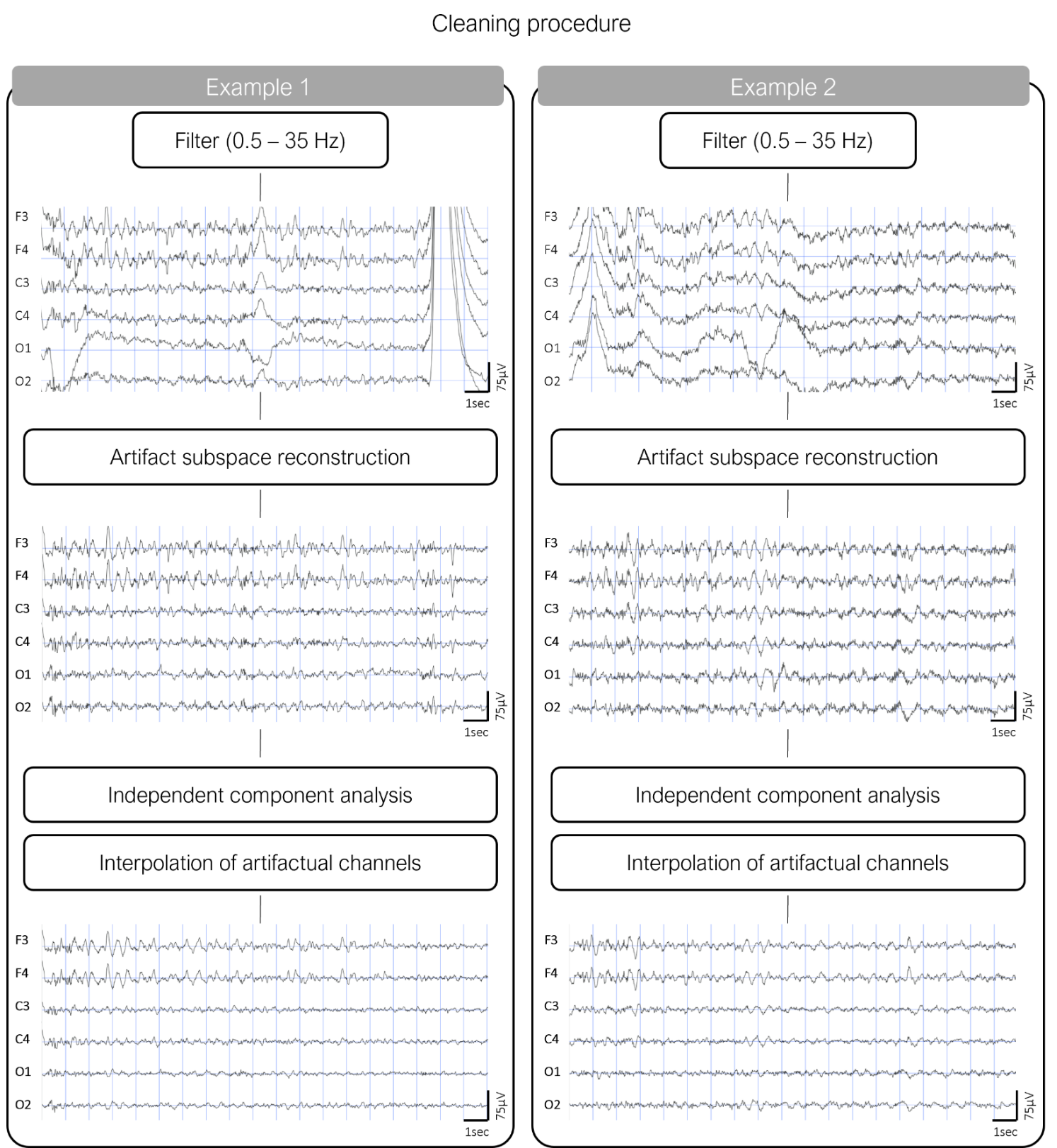


Fig. S8: Artifact removal procedure: Two examples of EEG traces of NREM parasomnia episodes illustrating the main steps of the procedure. A selection of 6 channels (F3, F4, C3, C4, 01, 02) referenced to the linked mastoid is shown.

Table S1

|  | Formula | Statistics | p |
| --- | --- | --- | --- |
| 1 | Prov/spont ~ surprise level + (1\|sub) | *X* ^2^(1) = 0.219 | 0.639 |
| 2 | CE/NE ~ prov/spont + (1\|sub) | *X* ^2^(1) = 1.274 | 0.264 |
| 3 | CE/CEWR ~ prov/spont + (1\|sub) | *X* ^2^(1) < 0.01 | 0.996 |
| 4 | CE/NE ~ BL/REC + (1\|sub) | *X* ^2^(1) = 0.828 | 0.362 |
| 5 | CE/CEWR ~ BL/REC + (1\|sub) | *X* ^2^(1) = 1.277 | 0.445 |
| 6 | CE/NE ~ time since lights off + (1\|sub) | *X* ^2^(1) = 0.582 | 0.784 |
| 7 | CE/CEWR ~ time since lights off + (1\|sub) | *X* ^2^(1) = -0.997 | 0.319 |
| 8 | CE/NE ~ time after first sleep onset + (1\|sub) | *X* ^2^(1) = 0.333 | 0.739 |
| 9 | CE/CEWR ~ time after first sleep onset + (1\|sub) | *X* ^2^(1) = -0.935 | 0.349 |
| 10 | CE/NE ~ duration + (1\|sub) | *X* ^2^(1) = 1.832  Fig.1C | 0.067 |
| 11 | CE/CEWR ~ duration + (1\|sub) | *X* ^2^(1) = 0.063  Fig.1C | 0.546 |
| 12 | CE clear/NE ~ duration + (1\|sub) | *X* ^2^(1) = 2.24 | 0.024 |
| 13 | CE/NE ~ log (delta) + log (beta) + (1\|sub) + (1\|prov/spont) | Fig.2, Fig.S1 |  |
| 14 | CE/NE ~ slow wave parameter + (1\|sub) + (1\|prov/spont) | Fig.3, Fig.S4 |  |
| 15 | CCE/NE ~ log (delta) + log (beta) + (1\|sub) | Fig.4A |  |
| 16 | ICE/NE ~ log (delta) + log (beta) + (1\|sub) | Fig.4A |  |
| 17 | CCE/NE ~ slow wave parameter + (1\|sub) | Fig.4B |  |
| 18 | ICE/NE ~ slow wave parameter + (1\|sub) | Fig.4B |  |
| 19 | CE/CEWR ~ log (delta) + log (beta) + (1\|sub) + (1\|prov/spont) | Fig.5, Fig.S6 |  |
| 20 | CE/CEWR ~ slow wave parameter + (1\|sub) + (1\|prov/spont) | Fig.6, Fig.S7 |  |
| 21 | CEWR/NE ~ log (delta) + log (beta) + (1\|sub) + (1\|prov/spont) | Fig.S3 |  |

Table S1: Generalized linear mixed models used in this work, with reference to the figure displaying the results (if present). When consisting in a single value, statistics are reported in the column ‘statistics’ and their p-value in the column ‘p’. For the other analyses at the channel/voxel level, see Table S3. Statistics used in the models in lines 1 to 12 are two-sided, those used in lines 13 to 21 are one-sided.

Table S2

| Patient | Episode type | Report of experience | Observed behavior | Coherence |
| --- | --- | --- | --- | --- |
| P2 | S | I was telling you that I had a secret, that I knew how to fall asleep without making any sound, so as to trick the recording machines. | Turns to the wall and says “You cannot?”, then, while lying prone and leaning on her forearms, whispers unintelligibly for a long time, before looking around and saying "I need to stop talking" then smiles. | Yes |
| P2 | S | I had to save a cockroach, or ladybugs from dying, from gliding down the wall. | Calls for someone, touches the wall, looks into the space between the wall and the bed, as if something had fallen there, continues to search and call for help. | Yes |
| P2 | S | I wanted to take a shower. I heard someone talking and I asked whether this person was talking to me, that’s all. | Opens eyes, looks puzzled, looks to her feet, looks to the right and says "are you talking to me?", then says "No", shakes her head, lies back on pillow and closes eyes. | Yes |
| P4 | S | Cookies | Sits up, says something, points to something with the left hand, then talks very fast and unintelligibly, sighs (or laughs), pauses as if listened to someone, then talks again, as if in a conversation. | No |
| P12 | S | I was looking for my baby daughter, she wasn’t in the bed anymore, I thought she had fallen off the bed, I think I even screamed for help. | Suddenly sits up, touches wall with right hand, looks under the covers, then under the bed, and cries "Help!" twice. | Yes |
| P14 | S | A piece of furniture falling down | Lies on side, suddenly opens eyes with frightened expression, gasps and then lies back down, looks puzzled. | Yes |
| P17 | S | I was about to fall asleep. I saw something that made me startle. | Suddenly sits up in bed and looks around, seems puzzled. | Yes |
| P17 | S | I think I saw someone just in front of me and that made me startle. | Suddenly sits up in bed and looks around, seems puzzled. | Yes |
| P17 | S | Going to eat a pizza with brother. | Suddenly opens eyes looks around, seems puzzled. Scratches forehead close to electrodes. | No |
| P2 | P | I dreamt that there was a man, a nurse in the back of the room, on the right. He said that I should not pay attention to him. | Lifts head and says "Hm?" twice, looks to the far right of the room several times. | Yes |
| P2 | P | It is always about taxes, I haven’t slept these days, this will cost me a lot in terms of bills. | Opens eyes, looks to the right rapidly, lifts head and torso, touches her face and eyes, looks around puzzled. | No |
| P4 | P | Green pastures | Says ‘Hm?’, then ‘wait’, makes a hand gesture as if wanted to stop something, then talks unintelligibly. | No |
| P5 | P | I was in the train, seeing landscapes passing by. | Sits up, looks at cable box, readjusts cable above head. Makes smacking sounds with mouth. | No |
| P9 | P | Christmas. A candle on the tree, like a child would draw it. It was outside. I was doing something with the tree, then I turned around and startled when you talked to me. | After alarm sound opens eyes and looks around, then at the ceiling, then talks (appears to have an imaginary conversation). Startles when experimenter starts talking to her. | Yes |
| P14 | P | I thought I had lost the EEG net and the cables. | Suddenly opens eyes, seems puzzled and worried, says: I don’t have anything on me anymore? Do I? | Yes |

Table S2: examples of parasomnia experiences and associated behaviors. S=spontaneously occurring episode. P=provoked episode. Coherence= coherence between report and behavior, as judged by at least two raters.

Table S3

| Duration (s) | CE | CEWR | NE |
| --- | --- | --- | --- |
| Average | 31.5 | 24 | 18.18 |
| Std | 19.9 | 18.3 | 12.15 |
| Median | 28.3 | 17 | 17 |
| Min | 3 | 9 | 4 |
| Max | 108 | 76 | 40 |

Table S3: Parasomnia episode duration. Average, standard deviation (std), median, minimum (min) and maximum (max) values for episodes with report of conscious experience (CE), report of conscious experience without recall (CEWR) and report of no experience (NE). All values are expressed in seconds.

Table S4

|  | scalp:  CE/NE ~ power + (1\|sub) + (1\|prov_spont) | source:  CE/NE ~ power  + (1\|sub) + (1\|prov_spont) | scalp:  CE/CEWR ~ power + (1\|sub) +(1\|prov_spont) | source:  CE/CEWR ~ power + (1\|sub) +(1\|prov_spont) | scalp:  CCE/NE ~ power + (1\|sub) | scalp:  CEWR/NE ~ power + (1\|sub) +(1\|prov_spont) |
| --- | --- | --- | --- | --- | --- | --- |
| Delta sleep α = 0.05 | Fig. S1A c1(n = 20): 1.84 | Fig. 2A c1(n = 353): 1.84 | Fig. S6A c1(n = 4): 1.88 | Fig. 5A c1(n = 48): 1.86 | Fig. 4A c1(n = 4): 1.81 | Fig. S3A c1(n = 4): 1.86 |
| Beta sleep α = 0.05 | Fig. S1B c1(n = 38): 1.96 | Fig. 2B c1(n = 775): 1.9 | Fig. S6B c1(n = 3): 2.04 | Fig. 5B c1(n = 101): 1.85 | Fig. 4B c1(n = 5): 1.9 | Fig. S3B c1(n = 34): 1.96, c2(n = 3): 2.01 |
| Delta epi  α = 0.05 | NS | Fig. 2A c1(n = 301): 1.88 | Fig. S6A c1(n = 6): 1.84 c2(n = 39): 1.84 | Fig. 5A c1(n = 515): 1.93 | Fig. 4A c1(n = 33): 1.83 | NS |

Table S4: summary of the statistics for the significant clusters (electrodes/voxels) emerging from the generalized linear mixed models with power spectral density as fixed factor (one-sided statistics). Formula of the model is reported in the top row. For each time frame (sleep/episode) and cluster index (c1: cluster 1, c2: cluster 2 …), cluster size (n =) and T-Wald statistics (averaged across the voxels/electrodes of the cluster) are reported. NS = non-significant.

Table S5

| PSG-parameter | Average | Std |
| --- | --- | --- |
| Sleep latency (to N1, in min) | 15.35 | 14.25 |
| Recording time (min) | 469.44 | 42.59 |
| Time after sleep onset (min) | 453.14 | 42.40 |
| Wake after sleep onset (WASO) (min) | 29.08 | 31.65 |
| Total sleep time (TST) (min) | 424.02 | 38.17 |
| Sleep efficiency (%) | 93.19 | 6.00 |
| REM latency (min) | 110.55 | 36.65 |
| N1 (min) | 26.10 | 15.30 |
| N2 (min) | 230.86 | 38.98 |
| N3 (min) | 82.00 | 28.23 |
| N1 (% of TST) | 6.16 | 3.67 |
| N2 (% of TST) | 54.45 | 8.41 |
| N3 (% of TST) | 18.95 | 6.54 |
| REM (% of TST) | 20.11 | 4.95 |
| Arousal index (n/h) | 13.11 | 5.43 |
| Apnea-hypopnea index (AHI) (n/h) | 4.27 | 4.91 |
| Periodic limb movements index (PLMI) (n/h) | 3.06 | 6.16 |

Table S5: Sleep parameters obtained from clinical polysomnography (PSG) in 22 patients.

Text S1

Additional information about patients

Reported age of onset of parasomnia episodes was 9.1 ± 5.6 yrs (3-22). Mean self-reported frequency of parasomnia episodes was distributed as follows: ⁓once a month: 2 patients (9%), 2-3 times a month: 6 patients (27%); ⁓once a week: 6 patients (27%); 2-3 times a week 5 patients (23%) and almost every night: 3 patients (14%). All 22 patients (100%) had a history of confusional arousals. In addition, 13 patients (59%) had a history of both sleep terrors and sleepwalking, 6 patients (27%) only of sleepwalking and 2 patients only of sleep terrors (9%). A family history of NREM parasomnias was present in 12 patients (54%). Neurological and psychiatric comorbidities included migraine (n=1), sleep paralysis (n=1) idiopathic hypersomnia (n=1), a history of attention deficit hyperactivity disorder in childhood (n=1) and a history of febrile convulsions in childhood (n=1). Two patients had a periodic leg movements of sleep (PLMS) index greater than 15/h (23/h and 16.4/h), and five patients had an AHI index greater than 5/h, of which two had an AHI greater than 15/h (16.7/h and 15.5/h).

Text S2

These large fronto-central slow waves are reminiscent of so-called type I slow waves (including K-complexes), which are likely to be related to phasic activations of arousal systems ^2,3^ and differ in several aspects from so-called type II slow waves (including delta waves) that constitute the background EEG activity of slow wave sleep and account for changes in the posterior hot zone of dreaming^4^. The fact that the short and relatively stereotyped behaviors without report of consciousness were not associated with these cortical EEG changes could reflect a functional subcortico-cortical disconnection, and a predominantly subcortical generation of behavior, similar to orienting and threat-related defense responses that can occur without participation of the cortex ^5–8^.

References

1. Siclari, F., Baird, B., Perogamvros, L., Bernardi, G., LaRocque, J. J., Riedner, B., Boly, M., Postle, B. R. & Tononi, G. The neural correlates of dreaming. *Nat. Neurosci.* **20**, 872–878 (2017).

2. Siclari, F., Bernardi, G., Riedner, B. A., LaRocque, J. J., Benca, R. M. & Tononi, G. Two Distinct Synchronization Processes in the Transition to Sleep: A High-Density Electroencephalographic Study. *Sleep* **37**, 1621–1637 (2014).

3. Bernardi, G., Siclari, F., Handjaras, G., Riedner, B. A. & Tononi, G. Local and widespread slow waves in stable NREM sleep: Evidence for distinct regulation mechanisms. *Front. Hum. Neurosci.* **12**, 1–13 (2018).

4. Siclari, F., Bernardi, G., Cataldi, J. & Tononi, G. Dreaming in NREM sleep: A high-density EEG study of slow waves and spindles. *J. Neurosci.* **38**, 9175–9185 (2018).

5. Lee, K. H., Tran, A., Turan, Z. & Meister, M. The sifting of visual information in the superior colliculus. *Elife* **9**, e50678 (2020).

6. Krauzlis, R. J., Lovejoy, L. P. & Zénon, A. Superior colliculus and visual spatial attention. *Annu. Rev. Neurosci.* **36**, 165–182 (2013).

7. Kragel, P. A., Čeko, M., Theriault, J., Chen, D., Satpute, A. B., Wald, L. W., Lindquist, M. A., Feldman Barrett, L. & Wager, T. D. A human colliculus-pulvinar-amygdala pathway encodes negative emotion. *Neuron* **109**, 2404-2412.e5 (2021).

8. Liu, Y.-J., Wang, Q. & Li, B. Neuronal responses to looming objects in the superior colliculus of the cat. *Brain. Behav. Evol.* **77**, 193–205 (2011).
