# Supplementary material for: Shared EEG correlates between non-REM parasomnia experiences and dreams
Source: Nat Commun. 2024 May 9;15:3906. doi: 10.1038/s41467-024-48337-7 (PMC11082195; doi:10.1038/s41467-024-48337-7)
Supplement: Supplementary file 3 — Reporting Summary [file 41467_2024_48337_MOESM3_ESM.pdf]

nature portfolio

Corresponding author(s): Francesca Siclari
Last updated by author(s): 2024-28-2

Reporting Summary

Nature Portfolio wishes to improve the reproducibility of the work that we publish. This form provides structure for consistency and transparency in reporting. For further information on Nature Portfolio policies, see our Editorial Policies and the Editorial Policy Checklist.

Statistics

For all statistical analyses, confirm that the following items are present in the figure legend, table legend, main text, or Methods section.

n/a	Confirmed	
☐	☒	The exact sample size (*n*) for each experimental group/condition, given as a discrete number and unit of measurement
☐	☒	A statement on whether measurements were taken from distinct samples or whether the same sample was measured repeatedly
☐	☒	The statistical test(s) used AND whether they are one- or two-sided<br>*Only common tests should be described solely by name; describe more complex techniques in the Methods section.*
☐	☒	A description of all covariates tested
☐	☒	A description of any assumptions or corrections, such as tests of normality and adjustment for multiple comparisons
☐	☒	A full description of the statistical parameters including central tendency (e.g. means) or other basic estimates (e.g. regression coefficient) AND variation (e.g. standard deviation) or associated estimates of uncertainty (e.g. confidence intervals)
☐	☒	For null hypothesis testing, the test statistic (e.g. *F*, *t*, *r*) with confidence intervals, effect sizes, degrees of freedom and *P* value noted<br>*Give P values as exact values whenever suitable.*
☒	☐	For Bayesian analysis, information on the choice of priors and Markov chain Monte Carlo settings
☒	☐	For hierarchical and complex designs, identification of the appropriate level for tests and full reporting of outcomes
☒	☐	Estimates of effect sizes (e.g. Cohen's *d*, Pearson's *r*), indicating how they were calculated

Our web collection on statistics for biologists contains articles on many of the points above.

Software and code

Policy information about availability of computer code

Data collection	no code was used for data collection
Data analysis	Custom made code in Matlab, Mathworks (version 2015a) and R-Studio (version 1.1.463) are available at 'https://github.com/jcataldi2/SleepWalking/'.

For manuscripts utilizing custom algorithms or software that are central to the research but not yet described in published literature, software must be made available to editors and reviewers. We strongly encourage code deposition in a community repository (e.g. GitHub). See the Nature Portfolio guidelines for submitting code & software for further information.

Data

Policy information about availability of data

All manuscripts must include a data availability statement. This statement should provide the following information, where applicable:

- Accession codes, unique identifiers, or web links for publicly available datasets
- A description of any restrictions on data availability
- For clinical datasets or third party data, please ensure that the statement adheres to our policy

The EEG data generated in this study are provided in the Source Data file.

Research involving human participants, their data, or biological material

Policy information about studies with human participants or human data. See also policy information about sex, gender (identity/presentation), and sexual orientation and race, ethnicity and racism.

Reporting on sex and gender	Information about sex was collected on questionnaires and was self-reported, information about gender was not collected. Population is formed by 14 females and 8 males. Sex and gender-based analysis not applicable for this study.
Reporting on race, ethnicity, or other socially relevant groupings	Information on race, ethnicity or social grouping was not collected.
Population characteristics	Twenty-two patients with disorders of arousal were studied [14 females, aged 26.9 ± 5.3 yrs (average ± SD), range 18.3-36.3 yrs]. Patients who had presented at least one parasomnia episode per week during the last month were included. Exclusion criteria were major psychiatric or neurological comorbidities, medication (except birth control) and pregnancy. Thus, our findings may not be generalizable to other populations, including children.
Recruitment	Patients were recruited during clinical consultations at our sleep center, or by word of mouth.
Ethics oversight	The study was fully approved by the 'Commission cantonale d'éthique de la recherche sur l'être humain (CER-VD)'

Note that full information on the approval of the study protocol must also be provided in the manuscript.

Field-specific reporting

Please select the one below that is the best fit for your research. If you are not sure, read the appropriate sections before making your selection.

[x] Life sciences [ ] Behavioural & social sciences [ ] Ecological, evolutionary & environmental sciences

For a reference copy of the document with all sections, see nature.com/documents/nr-reporting-summary-flat.pdf

Life sciences study design

All studies must disclose on these points even when the disclosure is negative.

Sample size	The initial sample size calculation assumed that 14 subjects (70 to 80 parasomnia episodes in total) would yield a power of 0.95 for detecting differences of about 33% in measured variables (assuming an alpha level of 5%) (Julious 2004; Pockcock 1983). This estimation was based on the difference in cortical activation (ratio between low-frequency and high-frequency spectral power in the posterior hot zone at the source level) between consciousness and unconsciousness (62.2 mcV/Hz and 99.4 mcV/Hz, respectively, standard deviation of difference 27.0 mcV/Hz) obtained in seven healthy subjects undergoing serial awakenings NREM sleep (Siclari et al. 2017, study 2). However the number of subject was raised to 18 because the number of episodes that could be used for analysis was lower than predicted (see data exclusion below).
Data exclusions	4/22 subjects (presented no parasomnia episodes), 45/102 episodes because no consensus between raters or because no interview (see results).
Replication	This work replicated previous results obtained in a different study population (Siclari, Nature Neuroscience, 2017 and Siclari, Journal of Neuroscience, 2018).
Randomization	N/A as this was not a randomized study.
Blinding	Raters who judged parasomnia episodes were blind to the rating of the other raters.

Reporting for specific materials, systems and methods

We require information from authors about some types of materials, experimental systems and methods used in many studies. Here, indicate whether each material, system or method listed is relevant to your study. If you are not sure if a list item applies to your research, read the appropriate section before selecting a response.

Materials & experimental systems

n/a	Involved in the study
☒	☐ Antibodies
☒	☐ Eukaryotic cell lines
☒	☐ Palaeontology and archaeology
☒	☐ Animals and other organisms
☒	☐ Clinical data
☒	☐ Dual use research of concern
☒	☐ Plants

Methods

n/a	Involved in the study
☒	☐ ChIP-seq
☒	☐ Flow cytometry
☒	☐ MRI-based neuroimaging

Plants

Seed stocks	*Report on the source of all seed stocks or other plant material used. If applicable, state the seed stock centre and catalogue number. If plant specimens were collected from the field, describe the collection location, date and sampling procedures.*
Novel plant genotypes	*Describe the methods by which all novel plant genotypes were produced. This includes those generated by transgenic approaches, gene editing, chemical/radiation-based mutagenesis and hybridization. For transgenic lines, describe the transformation method, the number of independent lines analyzed and the generation upon which experiments were performed. For gene-edited lines, describe the editor used, the endogenous sequence targeted for editing, the targeting guide RNA sequence (if applicable) and how the editor was applied.*
Authentication	*Describe any authentication procedures for each seed stock used or novel genotype generated. Describe any experiments used to assess the effect of a mutation and, where applicable, how potential secondary effects (e.g. second site T-DNA insertions, mosiacism, off-target gene editing) were examined.*
